# Surface engineering of zinc phthalocyanine organic thin-film transistors results in part-per-billion sensitivity towards cannabinoid vapor

**DOI:** 10.1038/s42004-022-00797-y

**Published:** 2022-12-24

**Authors:** Zachary J. Comeau, Rosemary R. Cranston, Halynne R. Lamontagne, Cory S. Harris, Adam J. Shuhendler, Benoît H. Lessard

**Affiliations:** 1 Department of Chemical and Biological Engineering, University of Ottawa, 161 Louis Pasteur, K1N 6N5 Ottawa, ON Canada; 2 Department of Chemistry and Biomolecular Sciences, University of Ottawa, 150 Louis Pasteur, K1N 6N5 Ottawa, ON Canada; 3 Department of Biology, University of Ottawa, 30 Marie Curie, K1N 6N5 Ottawa, ON Canada; 4 University of Ottawa Heart Institute, 40 Ruskin St, Ottawa, K1Y 4W7 ON Canada; 5 School of Electrical Engineering and Computer Science, University of Ottawa, 800 King Edward Ave., K1N 6N5 Ottawa, ON Canada

**Keywords:** Sensors and biosensors, Electronic devices, Bionanoelectronics

## Abstract

Phthalocyanine-based organic thin-film transistors (OTFTs) have been demonstrated as sensors for a range of analytes, including cannabinoids, in both liquid and gas phases. Detection of the primary cannabinoids, Δ^9^-tetrahydrocannabinol (THC) and cannabidiol (CBD), is necessary for quality control and regulation, however, current techniques are often not readily available for consumers, industry, and law-enforcement. The OTFT characteristics, X-ray diffraction (XRD) spectra, and grazing incident wide angle x-ray scattering (GIWAXS) spectra of two copper and three zinc phthalocyanines, with varying degrees of peripheral fluorination, were screened to determine sensitivity to THC vapor. Unsubstituted ZnPc was found to be the most sensitive material and, by tuning thin-film morphology, crystal polymorphs, and thickness through altered physical vapor deposition conditions, we increased the sensitivity to THC by 100x. Here we demonstrate that deposition conditions, and the resulting physical film characteristics, play a significant role in device sensitization.

## Abstract (web summary)

Organic thin film transistors are potent sensors, but their charge transport layers (CTL) need to be carefully designed to reach desired analyte responses. Here, the authors show that zinc phthalocyanine sensors with a CTL thickness of 200 Å and low crystallinity are most susceptible to physical alterations upon exposure to analytes, resulting in large electrical changes and a low detection limit of 40 ppb for Δ9-tetrahydrocannbinol vapor.

## Introduction

Phthalocyanines (Pcs) and their derived metal complexes (MPcs) are macrocyclic organic compounds with a variety of industrial applications due to their useful spectral and electronic properties^1,2^. First reported in 1907, Pcs were highlighted for their excellent stability and brilliant color, finding extensive use as dyes and pigments^3–5^. As conjugated aromatic molecules, Pcs are often deposited as charge transport layers within organic thin-film transistors (OTFTs) and organic photovoltaics (OPVs)^6,7^. Pc-based OTFTs have been demonstrated as sensors for a variety of liquid and gas sensing applications^8–12^, including our groups’ recent demonstration of ratiometric detection and differentiation of Δ^9^-tetrahydrocannbinol (THC) and cannabidiol (CBD)^13,14^. The dominant active cannabinoids in *Cannabis sativa* smoke/vapour and consumer products, THC and CBD are used for therapeutic and recreational purposes^15,16^, however, as THC and CBD elicit different pharmacological effects, accurate, low-cost quantification and speciation is of interest to industry, law enforcement and consumers^17^. Quantitative commercial speciation can be accomplished with high-performance liquid chromatography (HPLC) or gas chromatography-mass spectrometry (GC-MS), though these techniques are often impractical for companies or individuals with limited resources. In our previous works^13,14,18^, we rationally examined a variety of single-use Pc-based OTFT sensors and established that the observed sensing responses were due to a combination of electrochemical interactions and physical effects on thin-film crystallinity. Ultimately, we established a relationship between OTFT sensing characteristics and analyte induced physical thin-film effects, which highlighted the importance of semiconducting material selection.

An advantage of Pcs is the ease by which modifications can be made to the central metal, peripheral, or axial substituents^2,7,19^, which allows tuning of the electronic, colorimetric, and solubility properties of the Pc to enable a range of applications. Peripheral *tetra*- (F_4_-MPc), *octa*- (F_8_-MPc), or *hexadeca*- (F_16_-MPc) Pc fluorination has been demonstrated for tuning thin-film band structures and crystal morphologies^20–22^. Increased fluorination was also found to confer increased solubility and n-type behavior in OTFTs^20,23,24^. With tuneable solubility and high stability, Pcs have been deposited as semiconducting layers for OTFTs both through solution techniques and physical vapor deposition (PVD)^7,22,25^. Examined with the goal of improving charge transport, deposition conditions generally focus on optimizing intermolecular distances while minimizing the negative effects of grain boundaries, interface energetics, and device architecture^22,26–28^. Electrically, improved charge transport is characterized by high mobility (*µ*), a voltage threshold (*V*_*T*_) near 0 V, low hysteresis, a large on/off current ratio (*On/Off*), and low defect density (*N*), all of which can be obtained from the transfer characteristics of an OTFT^7,29,30^. Post-deposition annealing can be accomplished with a range of techniques, most commonly heat or solvent vapor, but the goal of improved charge transport, and the mechanisms by which it’s achieved, remain the same^31,32^. Thus, in addition to molecular tuning, Pc thin films can be morphologically tuned through film engineering by altering the deposition surface, deposition conditions, or post-deposition annealing to obtain more favorable spectral characteristics or charge transport conditions^33,34^.

Post deposition annealing and sensing studies have highlighted zinc phthalocyanine (ZnPc) as a highly tuneable and sensitive material^24,35–38^ and we have previously demonstrated that analyte exposure induces structural changes within films, altering, and more often disrupting, charge transport pathways similarly to post deposition annealing techniques^18^. Thus, where analyte exposure can induce altered thin-film nanostructures triggering a larger sensing response, we hypothesize that structurally different films will have altered sensing responses. Here, we examine and screen the effects of THC vapor on thin-film crystallinity and OTFT performance of ZnPc, zinc tetrafluorophthalocyanine (F_4_-ZnPc), and zinc hexadecafluorophthalocyanine (F_16_-ZnPc), and compare the THC sensing performance to our previously evaluated copper phthalocyanine (CuPc), and copper hexadecafluorophthalocyanine (F_16_-CuPc) devices. We further investigate the most sensitive material by examining the effects of deposition conditions, crystal morphology, and thin-film thickness with the goal of increasing the sensitivity to THC vapor. By X-ray diffraction (XRD), grazing incident wide angle X-ray spectroscopy (GIWAXS), atomic force microscopy (AFM), and scanning electron microscopy (SEM), we interrogate thin-film surface nanostructures and morphology pre- and post-exposure to THC vapor, relating OTFT sensing characteristics to the physical characteristics of Pc thin films. Additionally, we perform real-time electrical characterization with vapor exposure to further elucidate the role of film thickness and polymorphism with sensing response. Ultimately, through material selection and tuned deposition conditions, we achieve a 100x increase in ZnPc device sensitivity to THC vapor, demonstrating the importance and utility of thin-film structures and engineering.

## Results and discussion

### Material selection

**Fig. 1:**
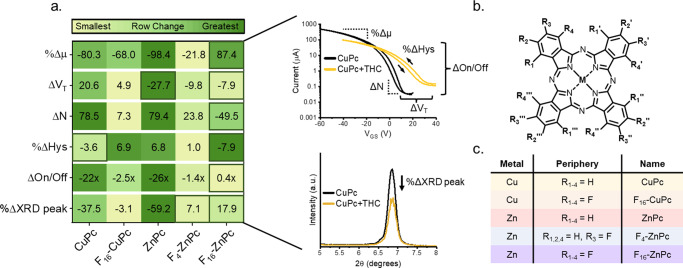
Effects of THC vapor on Pc OTFT electrical characteristics and XRD spectra. (**a**) CuPc, F_16_-CuPc, ZnPc, F_4_-ZnPc, or F_16_-ZnPc OTFTs were exposed to 4 ppm THC vapor over a period of 90 seconds. Boxed regions represent a sign change from the median response. Characteristic transfer and XRD spectra demonstrate sampled regions. Dashed lines represent regions of the curve within which slope was measured. (**b**) General Pc structure and (**c**) a table of the Pc’s studied. Mobility was calculated from the saturation region of the transfer curves while defect density and voltage threshold were estimated from the subthreshold slope, with average values taken from 20 devices.

CuPcs and ZnPcs, with various degrees of fluorination (CuPc, F_16_-CuPc, ZnPc, F_4_-ZnPc, F_16_-ZnPc) (Fig. 1), were deposited as 400 Å thin-films by PVD at 25 °C with a rate of 0.2 Å/s on OTS treated Si/SiO_2_ substrates with and without prepatterned gold electrodes. Pre-exposure GIWAXS and XRD spectra (Figure S1) revealed films with a high intensity (100) plane at *q* = ~0.50 Å^−1^ and a single XRD peak with *2θ* between 6–7°, suggesting a preferential orientation of the Pc ring to the substrate surface of ~75°; typical of α-crystal orientations with co-facial herringbone stacking^21,39^. Pre-exposure output and transfer curves (Figure S2) demonstrated OTFT performance in agreement with other sources^7,27,40,41^, with complete peripheral fluorination (F_16_-) of both CuPc and ZnPc conferring n-type OTFT behavior.

Pc thin-films were exposed to 4 ppm THC vapor over a period of 90 seconds and the percent change in peak mobility *(%Δµ*), voltage threshold shift (*ΔV*_*T*_), defect density (*ΔN*), hysteresis (*%ΔHys*), on/off current ratio (*ΔOn/Off*), and peak XRD intensity (*%ΔXRD*) was determined for each material and displayed in Fig. 1a. As previously established^18^, analytes, such as THC, when introduced to Pc thin films, in addition to the effects of intermolecular interactions, can also alter physical characteristics of the thin film, such as crystal packing and morphology, which can cause changes in OTFT performance. Here, with the exception of devices made with F_4_-ZnPc, the sign, and magnitude, of the change in XRD peak height correlates to the magnitude of the change in µ and *N*. By XRD and GIWAXS, the absence of any observed new crystal morphologies suggests that, without the formation of new polymorphs, mobility decreases are related to the degree of change in α-crystallinity of the bulk film. Limited changes in hysteresis and negligible OTFT bias stress effects suggests that exposure of OTFTs to THC vapor results in irreversible and relatively stable physical thin-film changes^29^.

In our previous works^14,18^, we found that THC, as a π-conjugated molecule with electron-donating properties^42^, interacts with the central metal of MPcs, and that these interactions are deferential to the species of the central metal. The molecular size, electronegativity, and valence structure of the central metal affects analyte interactions, which, in addition to electrochemical effects, can also result in physical effects, such as distortions to the Pc ring, and induce physical charge traps^38^. Here, CuPc and F_16_-CuPc-based devices both show + *ΔV*_*T*_ with exposure to THC, while the ZnPc, F_4_-ZnPc, and F_16_-ZnPc-based devices show *-ΔV*_*T*_, suggesting that the copper-THC interaction induces electron trapping while the zinc-THC interaction creates deep, but short life-time, hole traps^29,43^. The peripherally fluorinated Pcs demonstrate smaller changes in XRD peak intensity, *ΔN*, and *ΔOn/Off*, suggesting limitations to both metal-analyte interactions and film restructuring as a result of their increased size or peripheral electronegativity. With the exception of hysteresis, an overall comparison of the transfer curves and XRD spectra demonstrates that ZnPc devices undergo the greatest changes across all metrics with exposure to THC vapor. This further illustrates the critical impacts of analyte-Pc interactions and thin-film restructuring on over all device performance, highlighting the role of material selection in optimizing sensing responses.

### Effects of morphology

**Fig. 2:**
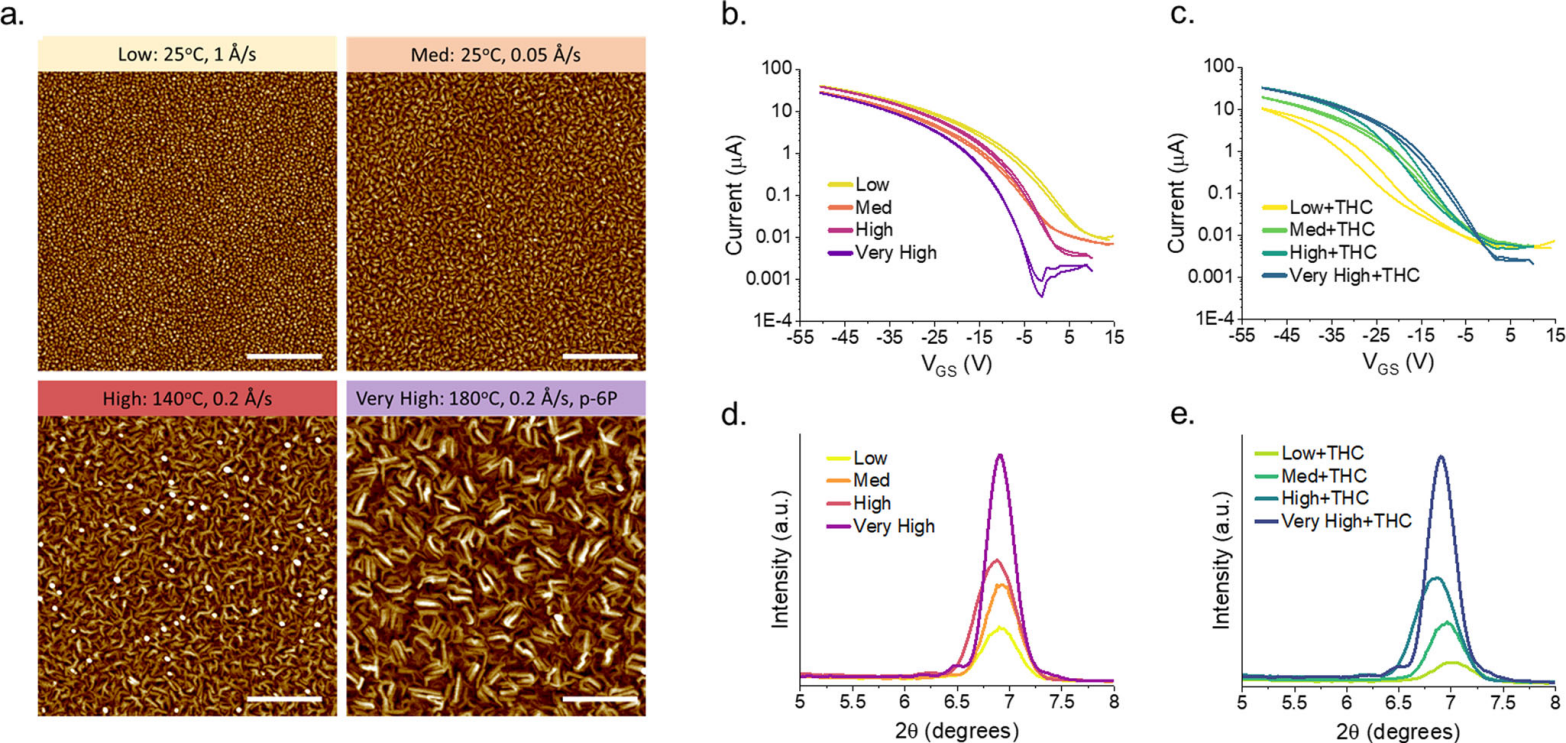
Effect of surface morphology on ZnPc OTFT sensitivity to THC vapor. (**a**) AFM images, transfer data, and XRD spectra of ZnPc OTFTs with varying degrees of crystallinity (**b**, **d**) pre- and (**c**, **e**) postexposure to 400 ppb THC vapor over 90 seconds. Low crystallinity thin-films were deposited at a rate of 1 Å/s and 25 °C, medium (med) at 0.05 Å/s and 25 °C, high at 0.2 Å/s and 140 °C, and very high at 0.2 Å/s and 180 °C with a pre-deposited monolayer of p-sexiphenyl (p-6P). Scale bars represent 500 nm.

With the greatest changes observed for ZnPc devices, we sought to further examine the effects of thin-film structures by preparing 400 Å α-ZnPc (*2θ* = 6.92°) OTFTs by altering the PVD rate or deposition substrate temperature, and altering the substrate surface by pre-depositing a monolayer of p-sexiphenyl (p-6P) as a patterning agent to afford varying degrees of α-crystallinity^44,45^. The fastest deposition rate (1 Å/s) at the lowest deposition substrate temperature (25 °C) resulted in small uniform grains by AFM, low peak intensity by XRD, and poor transfer characteristics (Fig. 2). Decreasing deposition rate, increasing deposition substrate temperature, or deposition on p-6P as a patterning agent, led to larger grain sizes, increased XRD peak intensity, and generally improved OTFT transfer characteristics (Table S1). A small peak, characteristic of β-ZnPc (*2θ* = 9.32°), was observed (Figure S3) for the depositions carried out at 180 °C, suggesting that some α- to β- phase transition was beginning to occur, however the relative intensity to the α- peak (*2θ* = 6.92°) was less than 2%.

Exposure of the least crystalline films to 4 ppm of THC vapor rendered the OTFTs inoperable. Films were instead exposed to 400 ppb THC vapor over a period of 90 seconds, inducing the greatest changes in the least crystalline films, and manifesting as *-%Δµ*, *-ΔV*_*T*_, an increase in hysteresis, decreased *On/Off*, and + *ΔN* (Table S1). Increased thin-film crystallinity reduced OTFT sensing responses, with the most crystalline films demonstrating limited changes in charge transport characteristics and negligible changes in XRD intensity. Measured by AFM, the surface area of each film was inversely proportional to the degree of alpha crystallinity, where the very high crystallinity films had the least surface area, while the low crystallinity films had the greatest surface area (very high = 6.43, high = 6.52, medium = 6.61, low = 6.81 µm of surface area in a 2.5×2.5 µm section). This suggests that smaller grains in the low crystallinity films affords increased surface area for ZnPc-THC interactions and intermolecular forces which facilitate greater changes by XRD and thus greater OTFT sensing responses^46^. Previously, we demonstrated a relationship between different Pcs, their analyte-induced physical film changes, and the measured OTFT sensing responses^18^. Here, these findings demonstrate that this relationship between physical thin-film changes and an OTFT sensor response can be applied to a single material, highlighting the importance of thin-film nanostructures for analytes-sensor interactions.

### Film thickness effects and real-time sensing

To assay the effects of thickness on the most sensitive thin-film conditions, we prepared OTFTs with 200 and 800 Å, low-crystallinity α-ZnPc films (deposition rate 1 Å/s at 25 °C) and exposed them to 40 ppb THC vapor over a period of 90 seconds (Figure S4). Pre-exposure, OTFTs of both thicknesses demonstrated similar transfer characteristics to low-crystallinity α-ZnPc 400 Å OTFTs, indicating that film thickness between 200–800 Å does not significantly influence the electrical performance of these low-crystallinity OTFTs. Compared to the 200 Å films, XRD spectra shows an expected, 59% more intense α- peak (*2θ* = 6.92°) for the 800 Å film, as the additional thickness provides additional diffraction signal^46^. The 2D scattering pattern determined by GIWAXS corroborates an α- phase orientation with a highly ordered (100) plane at *q* = 0.49 Å^−1^ and a preferential Pc orientation to the surface of ~74°. Exposure to THC vapor showed a similar + Δ*V*_*T*_, increase in hysteresis, -Δ*N*, and a decrease in XRD peak intensity for OTFTs of both thicknesses. The OTFTs with the thinnest, 200 Å films, in agreement with other works^47,48^, demonstrated a larger decrease in on-current, greater Δ*N*, and greater -%Δ*µ*, suggesting greater relative changes in film crystallinity, while changes in *V*_*T*_ and hysteresis suggest Pc-THC interactions which are independent of film thickness.

The initially screened thin-film conditions of 400 Å α-ZnPc OTFTs, deposited at a rate of 0.2 Å/s and 25 °C, demonstrate a negligible response when exposed to 40 ppb THC vapor (Figure S5). Comparing the response of the initially screened thin-film conditions to the sensitized response of the thinnest low crystallinity films, we demonstrate a 100x increase in post-exposure sensitivity to THC vapor. Thus, altered surface morphology and semiconductor thickness has a significant effect on OTFT sensitivity.

**Fig. 3:**
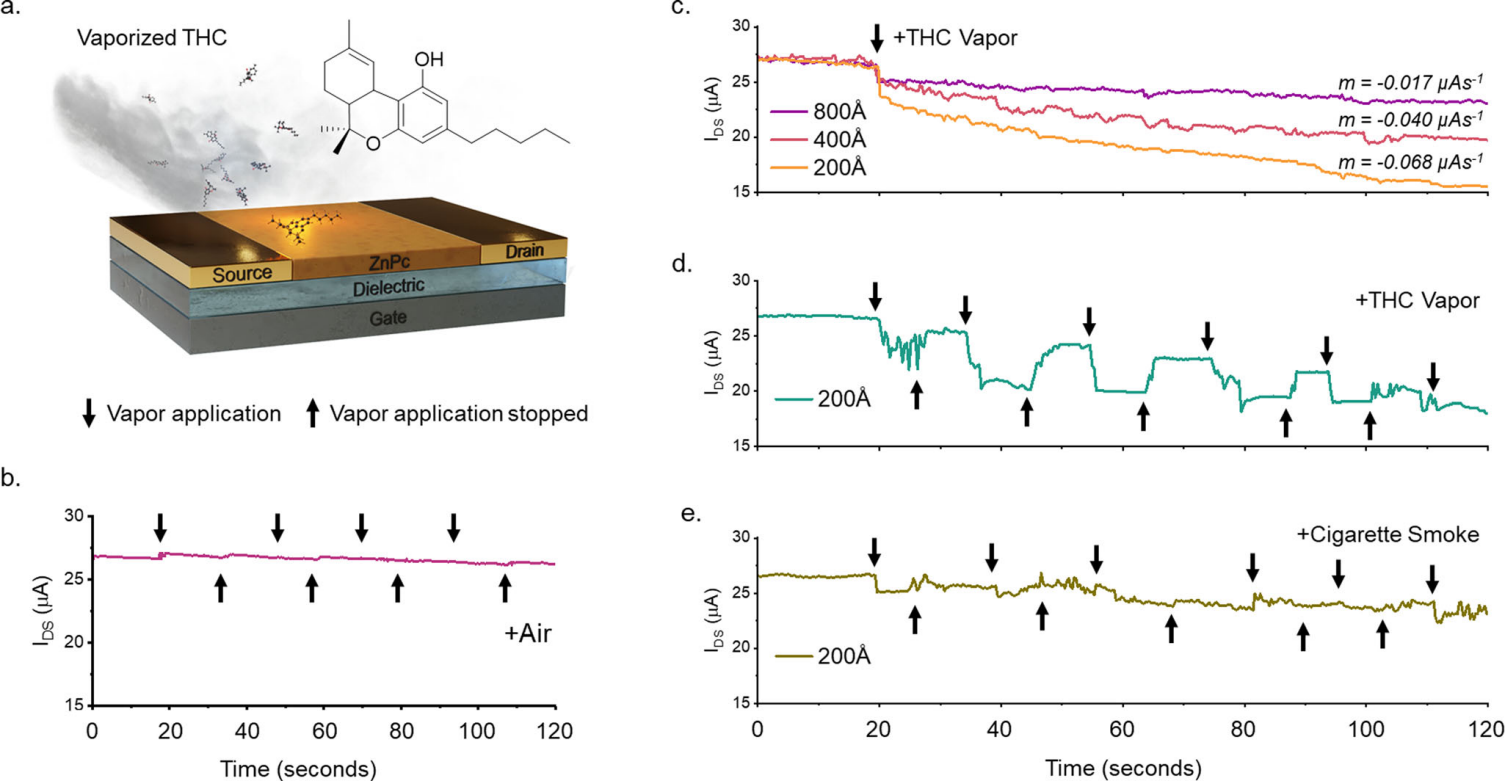
Real-time THC vapor exposure and detection. (**a**) Schematic showing the application of THC vapor to a ZnPc OTFT, (**b**) the effects of heated air, (**c**) the effects of continual THC vapor exposure on 200, 400, and 800 Å ZnPc OTFTs, (**d**) the effects of periodic THC exposure on a 200 Å ZnPc OTFT, and (**e**) the effects of 40 ppb cigarette smoke on 200 Å ZnPc OTFTs. A V_SD_ of −50 V was held and a V_GS_ of −40 V and was pulsed at a rate of 20 milliseconds on 80 milliseconds off over a period of 120 seconds while 40 ppb THC vapor was continually or periodically flowed over the surface of the OTFT in a 50 mL chamber. Slope was calculated from 20 to 120 seconds.

To examine the effects of THC exposure in real time, we operated low crystallinity α-ZnPc OTFTs at fixed saturation biases while they were continually exposed to 40 ppb THC vapor (Fig. 3). At operating biases of *V*_*SD*_ = −50 V and *V*_*GS*_ = −40 V, continuous THC vapor exposure caused an immediate, sharp, operating current decrease followed by a sustained decrease in operating current at a constant rate, which was mediated by thin-film thickness (Fig. 3c). Pulsed exposure demonstrated a similar effect (Fig. 3d and S6), where the onset of THC vapor induced an immediate decrease in operating current followed by a sustained, thickness mediated, decrease over time. However, after short exposures (~10 seconds), operating current recovered sharply when vapor application was stopped, though not to pre-exposure levels, and subsequent exposures reduced the magnitude of both the operating current decrease and recovery with vapor onset and offset.

The magnitude of the effects of vapor onset, continued exposure, and vapor offset were inversely related to film thickness, where the thinnest, 200 Å films, demonstrated the largest responses, consistent with OTFT sensors made with Cl-AlPc^47^. Additionally, for both pulsed and continual modes of exposure, after 90 seconds, the operating current is consistent with the operating current of films of the same thickness characterized post-exposure at the same bias conditions, suggesting prolonged exposure limits the observed recovery. Both periodic exposures to forced air and continued device operation in air (Fig. 3b), yielded negligible changes in operating current.

The observed, sharp, but semi-reversible, decreases in operating current with THC vapor onset suggests that the introduction of THC immediately causes hole-trapping effects, lowering the operating current. Sustained, irreversible decreases in operating current with continued THC vapor application then suggests that over time, irreversible structural defects occur within the film, permanently lowering operating current, and limiting repeated sensor use. That both the immediate and sustained decreases in operating current are thickness dependant supports these conclusions, as both the amounts of THC relative to Pc within the film and the THC-induced structural defects will be greatest for the thinnest films. Periodic exposure of the 200 Å films to 40 ppb cigarette smoke yields small onset current decreases with smoke application, slight current recovery when smoke application stops, and a slight decrease in current over time (Fig. 3e). However, the magnitude of the operating current decrease was small and the results noisy, indicating that other vaporized organic compounds have different effects on the electrical performance of the substrates, and demonstrating the innate selectivity of ZnPc for this application. Thus, every step of sensor manufacturing impacts device performance, from material selection, deposition conditions, and thin-film thickness.

### Effects of polymorphism

**Fig. 4:**
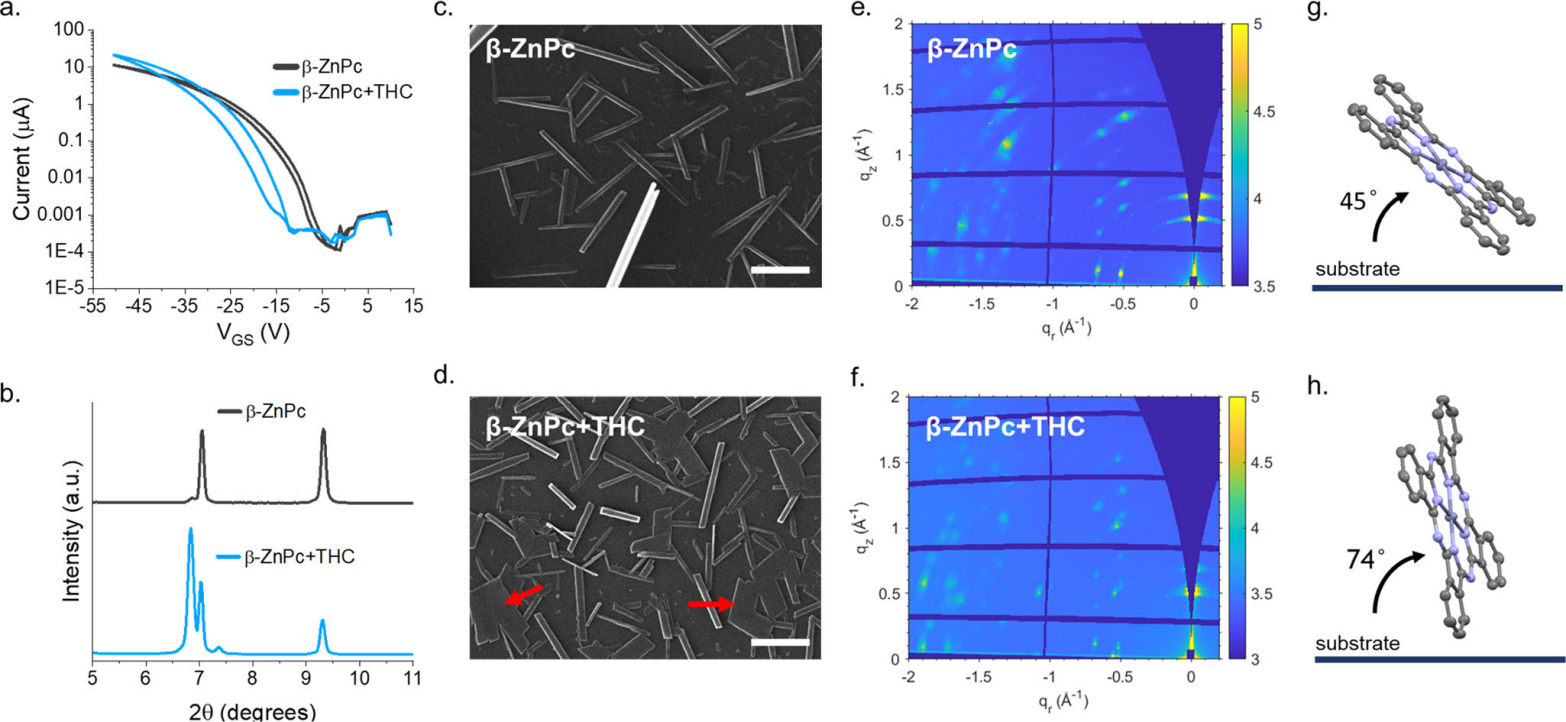
Effect of β- morphology on ZnPc OTFT sensitivity to THC vapor. (**a**) Transfer data, (**b**) XRD spectra, and (**c**, **d**) SEM images of ZnPc OTFTs after exposure to 400 ppb THC vapor over a period of 90 seconds. Scale bars represent 2 µm and red arrows denote sheet formation. (**e**, **f**) 2D scattering patterns (θ = 0.1°) of β-ZnPc thin films deposited on Si substrates and (**g**, **h**) a schematic showing GIWAXS determined preferential phase angle to the substrate pre- and post- exposure to THC vapor.

To analyze the effects of crystal polymorphism on sensing response, we prepared β-ZnPc thin-films by treating 400 Å α-ZnPc thin-films with toluene vapor for 24 h (Fig. 4) resulting in OTFTs with lower peak *µ*, lower *V*_*T*_, lower off current, and lower *N* in comparison to α-ZnPc OTFTs. Additionally, large, µm-scale rectangular crystals are observable by SEM. XRD spectra of the toluene-treated films showed two sharp peaks of equal intensity at *2θ* = 7.04 and 9.32°, consistent with reported β-Pc morphology^31^, and a small shoulder peak at *2θ* = 6.84°. The azimuthally-integrated GIWAXS pattern of the films is in good agreement with the β-ZnPc pattern predicted by single crystal XRD (Figure S7) confirming complete transition from α- to β- phase. The 2D scattering pattern reveals a highly ordered crystalline thin film with the (100) plane at *q* = 0.50 and the (10-2) plane at *q* = 0.68 Å^−1^ along the *q*_*z*_ axis suggesting multiple molecular orientations however with a preference of the Pc ring aligned ~45° to the surface.

Exposure to 400 ppb THC vapor over a period of 90 seconds resulted in a 10.3 µA increase in peak operating current, a −7.4 V in *ΔV*_*T*_, and a 4.1 V increase in hysteresis. By XRD, the intensity of the peak at *2θ* = 7.04° does not change, while the intensity of the peak at *2θ* = 9.32° decreases by 53%. The shoulder peak at *2θ* = 6.84° increases in intensity by 20-fold, and a new peak at *2θ* = 7.38° appears, indicating morphological changes. Visualized by SEM, previously well-ordered, narrow, rectangular crystals become broad and sheet-like post-exposure (red arrows). By GIWAXS we observe a decrease in relative peak intensity corresponding to the (10-2) plane (*q* = 0.68 Å^−1^) and peak splitting at *q* = ~0.50 Å^−1^, corresponding to the (100) plane (Figure S7) in β-ZnPc thin films post-exposure to THC vapor. Additionally, the 2D scattering pattern of post-exposure films exhibit a narrowing of the partial arcs and a change in preferential ZnPc orientation to the surface from ~45° to ~74°.

β-ZnPc, despite being highly crystalline, generally demonstrates poor semiconducting performance due to suboptimal charge transport distances caused by close intermolecular spacing and an increased face-on configuration^49^. Thus, the observed increase in operating current as an OTFT “turn-on” response may be a result of partial conversion of β-ZnPc crystals to an α-, or an α-like, polymorph through exposure to THC vapor and suggests the interstitial incorporation of THC into the ZnPc crystallites. Low defect density (*N* = 5.4•10^−12^), extracted from the subthreshold swing, post-exposure suggests the sheet-like structures provide excellent charge transport pathways, with hysteresis possibly resulting from the large interfacial areas between the sheets^22,50^. A new XRD peak at *2θ* = 7.38° in the post-exposure β-ZnPc films, but not in the post-exposure α- films or the pre-exposure β- films, suggests a tertiary morphology in the THC vapor induced phase transition of β-ZnPc^31^.

Real-time characterization of the β-ZnPc OTFTs with continual 400 ppb THC vapor exposure (Figure S8) demonstrated a turn-off response when operated with *V*_*SD*_ of −50 V and *V*_*GS*_ of −20 V. Like the α-ZnPc OTFTs, with the onset of THC vapor there is an immediate decrease in operating current, however, rather than a subsequent sustained decrease with constant slope, instead there is a rapid decrease in operating current with a decaying slope. As a large *V*_*T*_ shift is observed for the β-ZnPc OTFTs characterized post-exposure, a decrease in operating current is expected at these bias conditions. Inconsistencies in the pre-exposure operating current and vapor onset effects for the β-ZnPc OTFTs, in comparison to the α-ZnPc OTFTs, are likely a result of uneven distribution and orientation of the β-crystals between OTFT electrodes.

With a *V*_*GS*_ bias of −40 V, there is again an immediate decrease in OTFT operating current with the onset of THC vapor with limited change for ~40 seconds, whereupon the operating current begins to fluctuate and trend upwards. Comparing to periodic exposure with the same bias conditions, we observe a similar fluctuating climb in operating current after ~35 seconds of exposure to THC vapor. Fluctuations in operating current suggest that significant µm-scale recrystallization events begin after ~35–40 seconds of exposure, in comparison to the immediate sustained decrease in operating current observed for the α-ZnPc OTFTs. Moreover, sharp decreases in operating current with vapor onset, and recovery with vapor offset, again suggests THC is introducing or acting as a hole-trap within the film. Additionally, where the most crystalline α-ZnPc thin-films demonstrated the least OTFT and XRD changes, highly crystalline β-ZnPc films demonstrated significant re-crystallization with exposure to THC vapor. Thus, the degree of molecular order on the surface is not necessarily indicative of either sensing response or the analyte-induced physical effects. Instead, the lowest crystallinity α-ZnPc OTFTs demonstrated the greatest electrical sensitivity to THC vapor, exhibiting the balance between thin-film morphology, recrystallization, and surface area for intermolecular interactions.

## Conclusions

The effects of THC vapor exposure on both the central metal and peripheral fluorination demonstrated that non-fluorinated ZnPc OTFTs exhibited the greatest electrical and structural sensitivity compared to isostructural CuPc OTFTs and devices. Increasing peripheral fluorination was found to limit analyte-induced structural changes while the central metal-mediated voltage threshold shifts. Preparing α-ZnPc films with varying degrees of crystallinity revealed that the least crystalline films were the most susceptible to physical alterations upon exposure to analytes and had the largest OTFT electrical changes. Film thickness was also found to effect sensitivity, with 200 Å, low crystallinity films demonstrating sensitivity to 40 ppb THC vapor. Real-time characterization of low crystallinity α-ZnPc OTFTs demonstrated an immediate, reversible hole-trapping effect with the onset of THC vapor and a sustained, irreversible operating current decrease due to physical film restructuring. In contrast, highly crystalline β-ZnPc films demonstrated significant structural changes when exposed to THC vapor. Such films transitioned from large, regular, μm-scale crystals to more sheet-like structures, with a change in preferred substrate angle from 45 to ~74° and demonstrating a turn-on OTFT response. Through film engineering of Pc-based OTFT sensors we achieved a 100x increase in sensitivity over our previously developed CuPc-based devices^13^, illustrating the importance of not only material selection, but also thin-film nanostructures, thickness, and polymorphism in Pc-OTFT sensor implementations.

## Experimental

### Materials

CuPc (copper(II) phthalocyanine), F_16_-ZnPc (zinc 1,2,3,4,8,9,10,11,15,16,17,18,22,23,24,25-hexadecafluoro phthalocyanine), and tricholoro(octyl)silane were obtained from Sigma Aldrich. F_16_-CuPc (copper(II) 1,2,3,4,8,9,10,11,15,16,17,18,22,23,24,25-hexadecafluoro phthalocyanine), and ZnPc (zinc phthalocyanine) were obtained from TCI chemicals. F_4_-ZnPc was synthesized according to ref. ^35^ and confirmed by mass spectrometry. All Pcs were purified by train sublimation prior to use. Cannabinoid standards were obtained from Toronto Research Chemicals. All solvents were HPLC grade and purchased from Fischer Scientific.

### Thin film fabrication

Si substrates with 230 nm thermally grown SiO_2_ dielectric and prepatterned gold source-drain electrodes (W = 2000 μm, L = 10 μm), were purchased from Fraunhofer IPMS and used to fabricate bottom-gate bottom-contact (BGBC) transistors. Si substrates with 300 nm thermally grown SiO_2_ were purchased from Ossila and used for fabrication of thin films for XRD and GIWAXS. Wafers were prepared as described in ref. ^18^ before being transferred to an Angstrom EvoVac thermal evaporator where deposition rate and thin-film thickness was controlled by quartz crystal microbalance and substrate temperature was controlled by thermocouple.

β-ZnPc films were generated by exposing 400 Å ZnPc films deposited at 25 °C at a rate of 0.2 Å/s to 50 °C toluene vapor in a custom vapor chamber for 24 h followed by a baking at 70 °C for 45 minutes in a vacuum oven.

### Vapor exposure

THC was dissolved in methanol and loaded on to a steel wool frit where it was allowed to dry before being placed in a Volcano Medic vaporizer set to 210 °C temperature. An 8 L balloon was filled completely before being evacuated into a 50 mL volume vapor chamber in which the substrates were placed.

### OTFT characterization

Organic thin film transistors were characterized by applying a voltage bias between gate and source electrodes with BeCu alloy probe tips and the source drain current (*I*_*SD*_) was measured with a Keithley 2614B on a custom electrical probe station. Mobility was calculated using the following:

$${I}_{{DS}}=\frac{\mu {C}_{I}W}{2L}{({V}_{{GS}}-{V}_{T})}^{2}$$

Where *C*_*i*_ is the dielectric capacitance, *W* and *L* are the width and length of the semiconducting channel, and *V*_*T*_ describes the threshold voltage at which *I*_*DS*_ begins to rapidly increase. To mitigate the effects of bias stress, the gate bias was pulsed at 20 millisecond intervals with an 80 millisecond delay. To saturate the devices, six transfer curves were obtained for each device with the final three averaged to yield a characteristic transfer curve. Defect density (N) was determined with the following:

$$N=\left(\frac{{Sq}}{{kTlin}(10)}-1\right)\frac{{C}_{i}}{q}$$

Where *S* is the subthreshold slope, as estimated graphically from the transfer data, *q* is the electronic charge, *k* is Boltzmann’s constant, and *T* is temperature.

### XRD and GIWAXS

XRD characterization was performed using a Rigaku Ultima IV powder diffractometer with a Cu-Kα (λ = 1.5418 Å) source. Thin-film substrate measurements were taken with a scan range of 5° < 2θ < 11° at a rate of 0.5° min^−1^ with a spin rate of 30 rpm.

GIWAXS experiments were performed at the Canadian Light Source (CLS) in Saskatoon, Canada using the Brockhouse Diffraction Sector (BXDS) beamline with a photon energy of 15.1 keV, and the SOLEIL Synchrotron facility in Saint-Aubin, France using the SIRIUS beamline with a photon energy of 10 keV. For data collected at CLS, GIWAXS patterns were collected using a Rayonix MX300 CCD detector (73.242 μm x 73.242 μm pixel size), placed 416 mm from the sample with an angle of incidence of θ = 0.3°. For data collected at SOLEIL, GIWAXS patterns were collected using a PILATUS3 S 1 M detector (172 μm x 172 μm pixel size), placed 330 mm from the sample with an angle of incidence of θ = 0.1°. All GIWAXS data was calibrated against a silver behenate standard and a poly(3-hexylthiophene-2,5-diyl) standard and analyzed using the GIXSGUI software package in MATLAB, where both polarization and solid-angle corrections were applied^51^.

### AFM and SEM

SEM measurements were performed with a Tescan Vega II on thin-film Pc devices at 20 kV. AFM measurements were performed with a Bruker Dimension Icon AFM, equipped with ScanAsyst-Air tips, and images were processed with NanoScope Analysis v.1.8. Scans were performed at a rate of 0.814 Hz with multiple sites investigated.

## Supplementary information

Supplementary Information

## Data Availability

All data is available upon request to the corresponding authors.
