# Supplementary material for: Surface engineering of zinc phthalocyanine organic thin-film transistors results in part-per-billion sensitivity towards cannabinoid vapor
Source: Commun Chem. 2022 Dec 24;5:178. doi: 10.1038/s42004-022-00797-y (PMC9814745; doi:10.1038/s42004-022-00797-y)
Supplement: Supplementary file 1 — Supplementary Information [file 42004_2022_797_MOESM1_ESM.pdf]

# Supplemental Information

# Surface engineering of zinc phthalocyanine organic thin-film transistors results in part-per-billion sensitivity towards cannabinoid vapor

Zachary J. Comeau[1,2], Rosemary R. Cranston[1], Halynne Lamontagne[1,2], Cory S. Harris[2,3], Adam J. Shuhendler[2,3,4]* and Benoît H. Lessard[1,5]*

*[1]Department of Chemical and Biological Engineering, University of Ottawa, 161 Louis Pasteur, Ottawa, ON, Canada, K1N 6N5*
*[2]Department of Chemistry and Biomolecular Sciences, University of Ottawa, 150 Louis Pasteur, Ottawa, ON, Canada K1N 6N5*
*[3]Department of Biology, University of Ottawa, 30 Marie Curie, Ottawa, ON, Canada K1N 6N5*
*[4]University of Ottawa Heart Institute, 40 Ruskin St, Ottawa, ON, Canada K1Y 4W7*
*[5]School of Electrical Engineering and Computer Science, University of Ottawa, 800 King Edward Ave. Ottawa, ON, Canada, K1N 6N5*

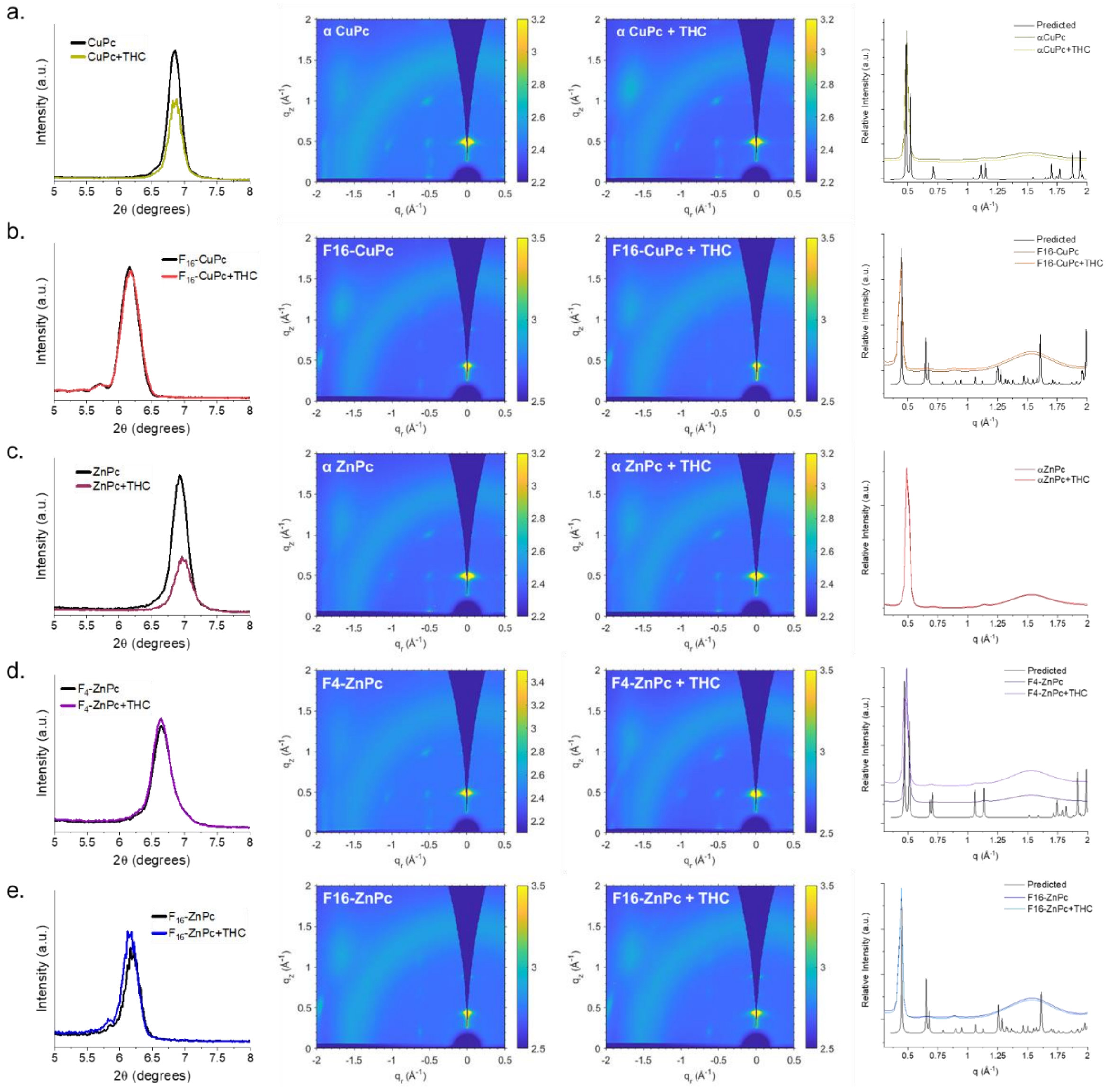


Figure S1. XRD, 2D scattering patterns (θ = 0.3°), and diffraction patterns predicted from single crystal and determined by GIWAXS of pre- and post- exposure films to THC vapor (a) α-CuPc (CCDC #219250) [1], (b) $F_{16}$-CuPc (CCDC #698474) [2], (c) α-ZnPc, (d) $F_4$-ZnPc (CCDC #1818040)[3], and (e) $F_{16}$-ZnPc (CCDC #1013162)[4].

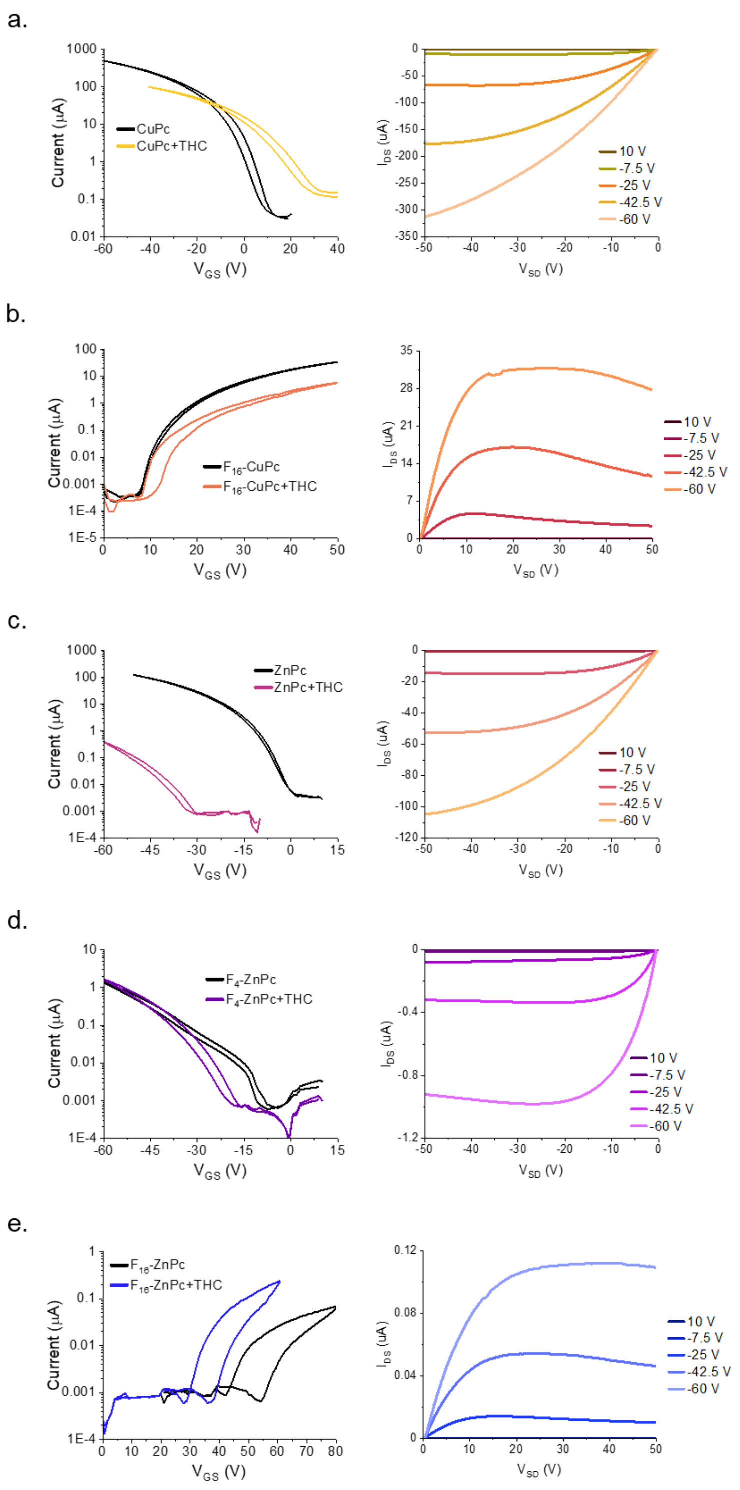


Figure S2. Characteristic transfer and output curves of Pc thin-films pre- and post- exposure to THC vapor. Twenty (a) CuPc, (b) $F_{16}$-CuPc, (c) ZnPc, (d) $F_4$-ZnPc, and (e) $F_{16}$-ZnPc OTFTs were characterized and then exposed to 4 ppm THC vapor over a period of 90 seconds.

Table S1. Calculated transfer data pre- and post- THC vapor exposure for morphologically different films[a]

| | Low | | Med | | High | | Very High | |
|---|---|---|---|---|---|---|---|---|
| | Baseline | +THC | Baseline | +THC | Baseline | +THC | Baseline | +THC |
| Peak Mobility [$10^{-2}$ $cm^2/Vs$] | 2.5±0.2 | 1.8±0.3 | 2.4±0.1 | 2.0±0.2 | 2.9±0.2 | 2.6±0.2 | 2.7±0.1 | 2.7±0.1 |
| Voltage Threshold [V] | -2.8±0.4 | -19.3±2.1 | -8.2±0.7 | -12.6±0.8 | -6.8±1.0 | -8.9±0.8 | -11.4±1.1 | -8.2±1.2 |
| Defect Density [$10^{12}/cm^2V$] | 12.3±1.3 | 21.4±2.3 | 13.7±0.8 | 15.4±1.0 | 7.51±0.5 | 12.5±0.8 | 4.23±0.4 | 9.9±0.7 |
| Maximum Hysteresis [V] | 1.6 | 6.1 | 1.3 | 2.5 | 1.1 | 3.2 | 0 | 2.1 |
| On/Off Ratio | $4 \cdot 10^3$ | $2 \cdot 10^3$ | $4 \cdot 10^3$ | $4 \cdot 10^3$ | $1 \cdot 10^4$ | $7 \cdot 10^3$ | $7 \cdot 10^4$ | $2 \cdot 10^4$ |

a. Mobility, voltage threshold, and defect density was calculated from the transfer data of 18 OTFTs for each condition and averaged.

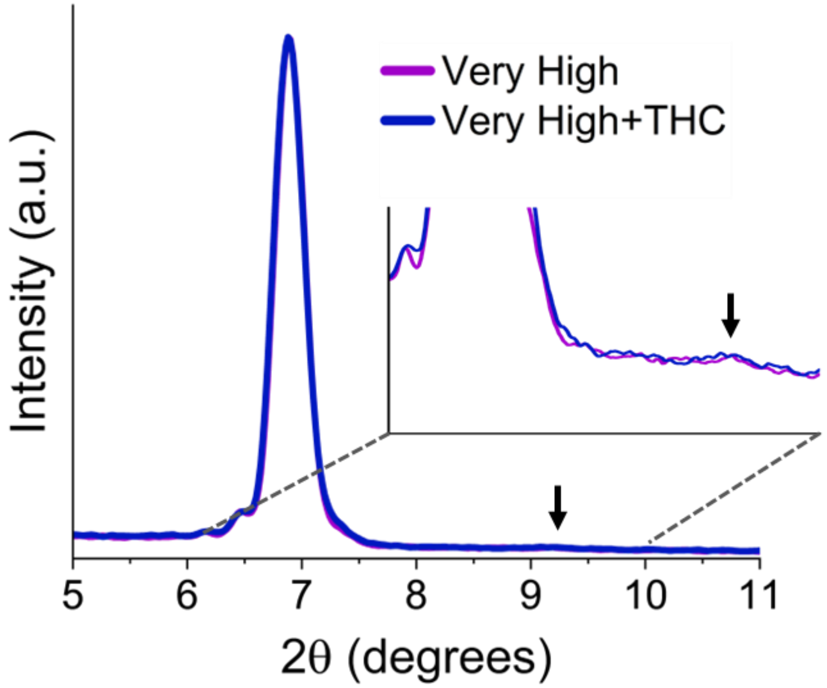


Figure S3. XRD spectra of very high crystallinity ZnPc thin-films pre- and post-THC vapor exposure, with inset showing spectra between 6 and 10 degrees 2θ. ZnPc thin-films were deposited at a rate of 0.2 Å/s and 180°C with a pre-deposited monolayer of p-sexiphenyl (p-6P) before being exposed to 400 ppb THC vapor over 90 seconds.

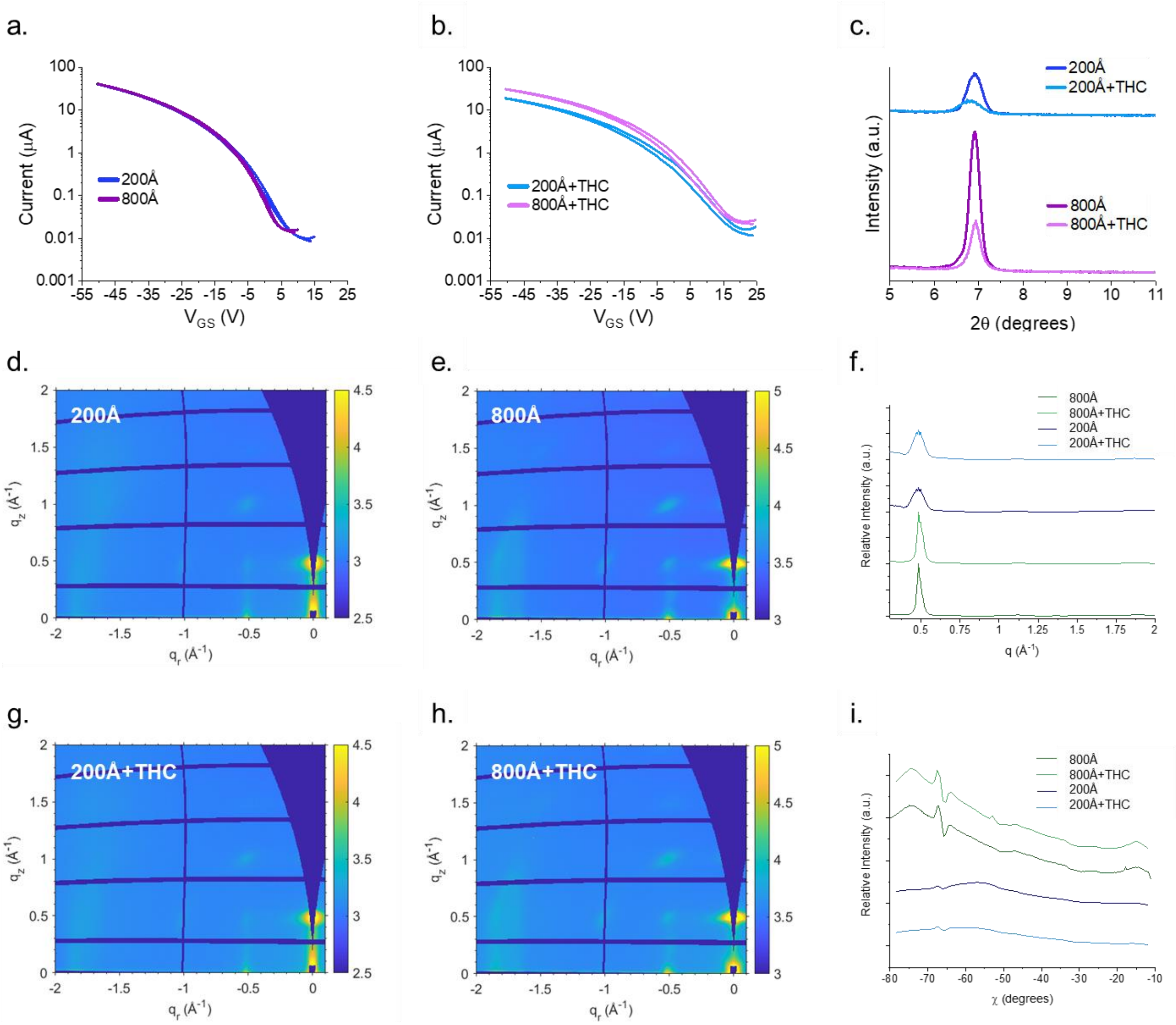


Figure S4. Effects of thickness on THC vapor sensitivity of ZnPc thin films. Transfer curves of 200 and 800 Å ZnPc OTFTs (a) pre- and (b) post- THC vapor exposure. (c) XRD spectra of thin-films pre- and post-exposure.2D scattering patterns (θ = 0.1°) of (d, g) 200 and (e, h) 800 Å α-ZnPc pre- and post- exposure to THC vapor. (f) Diffraction patterns determined by GIWAXS and (i) linecut profiles with respect to χ using a *q* range between 1.9-2.1 $Å^{-1}$.

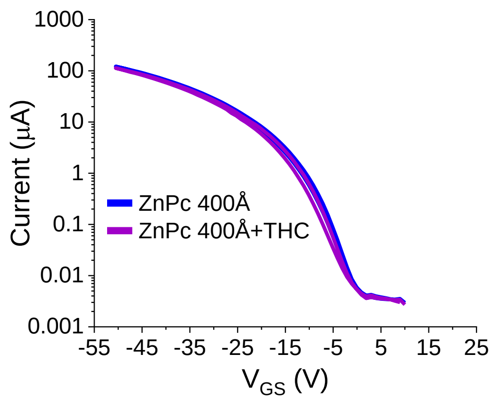


Figure S5: Initially screened ZnPc OTFT transfer curves pre- and post- exposure to 40 ppb THC vapor for 90 seconds. Films were deposited at a rate of 0.2 Å/s.

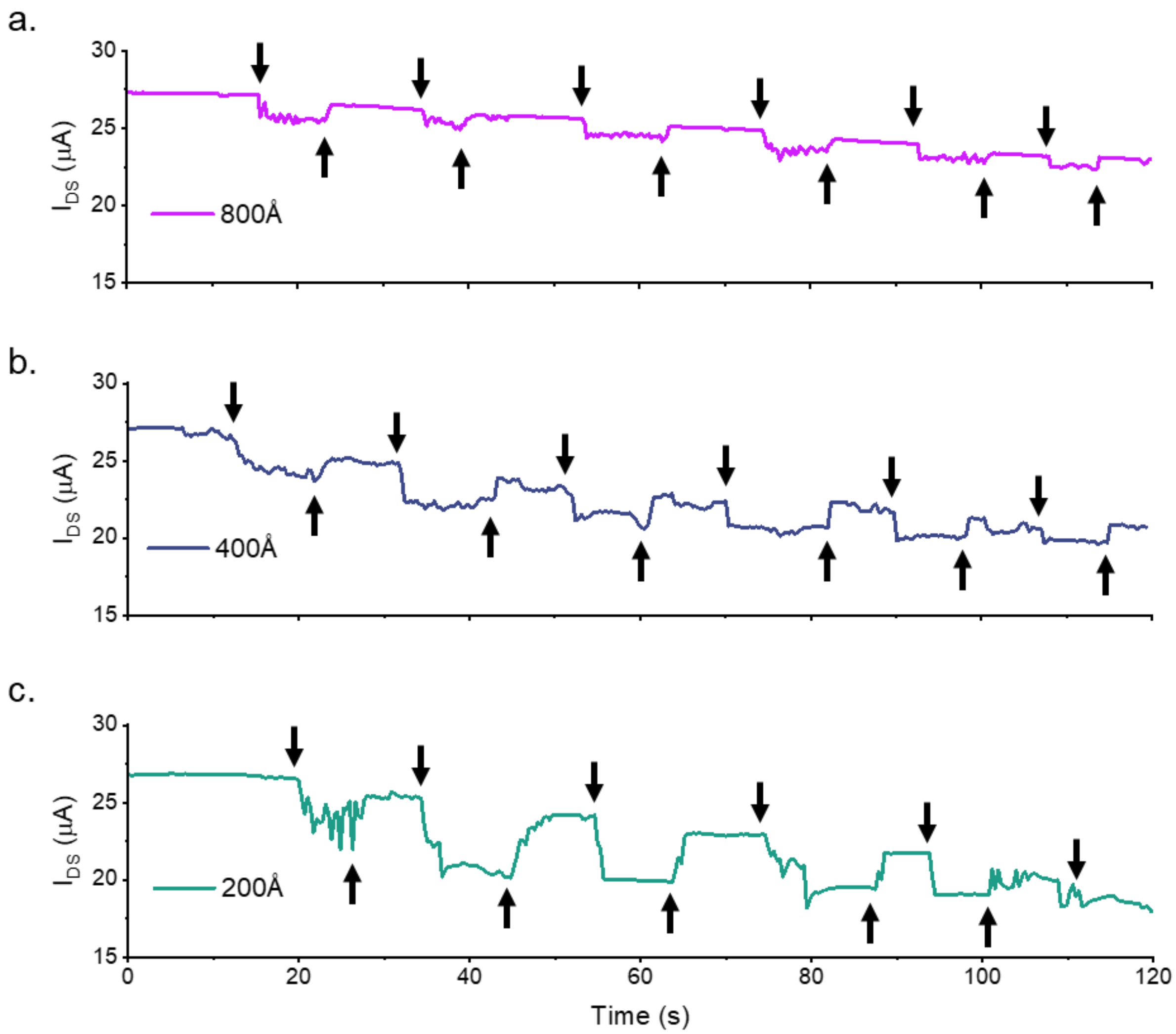


Figure S6. Periodic in-situ THC vapor exposure and detection. The effects of periodic THC exposure on (a) 800, (b) 400, and (c) 800 Å ZnPc OTFTs. A $V_{SD}$ of -50 V was held and a $V_{GS}$ of -40 V and was pulsed at a rate of 20 milliseconds on 80 milliseconds off over a period of 120 seconds. 40 ppb THC vapor was introduced to a 50 mL chamber.

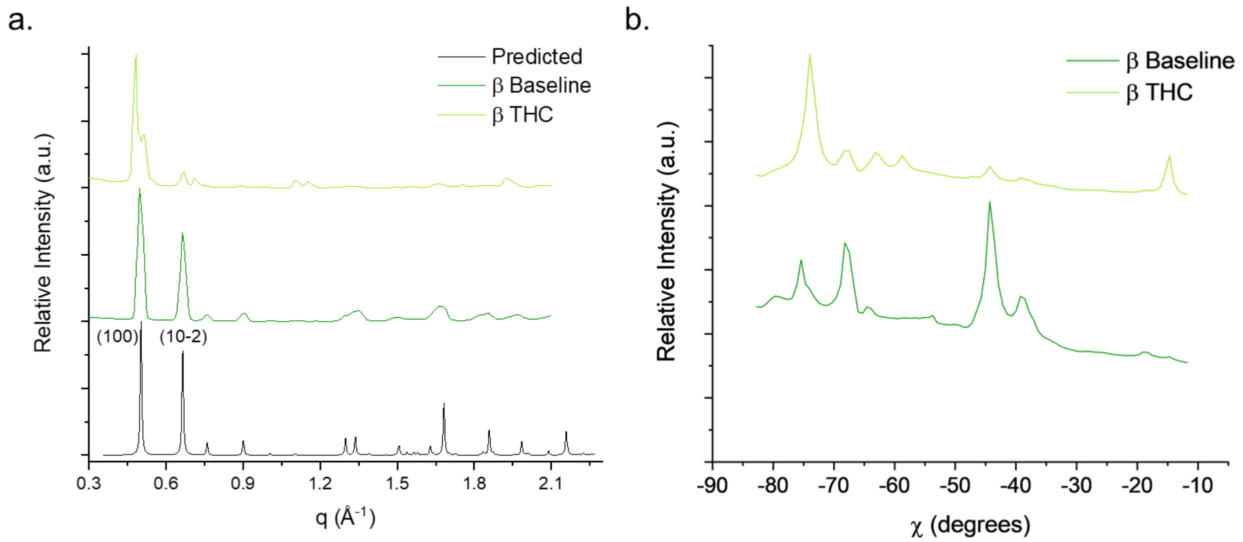


Figure S7. (a) Diffraction pattern of β-ZnPc predicted from single crystal (CCDC #2098417)[3] and β-ZnPc pre- and post- exposure to THC vapor determined by GIWAXS. (b) Linecut profiles of pre- and post- exposed β-ZnPc with respect to χ using a *q* range between 1.9-2.1 Å$^{-1}$.

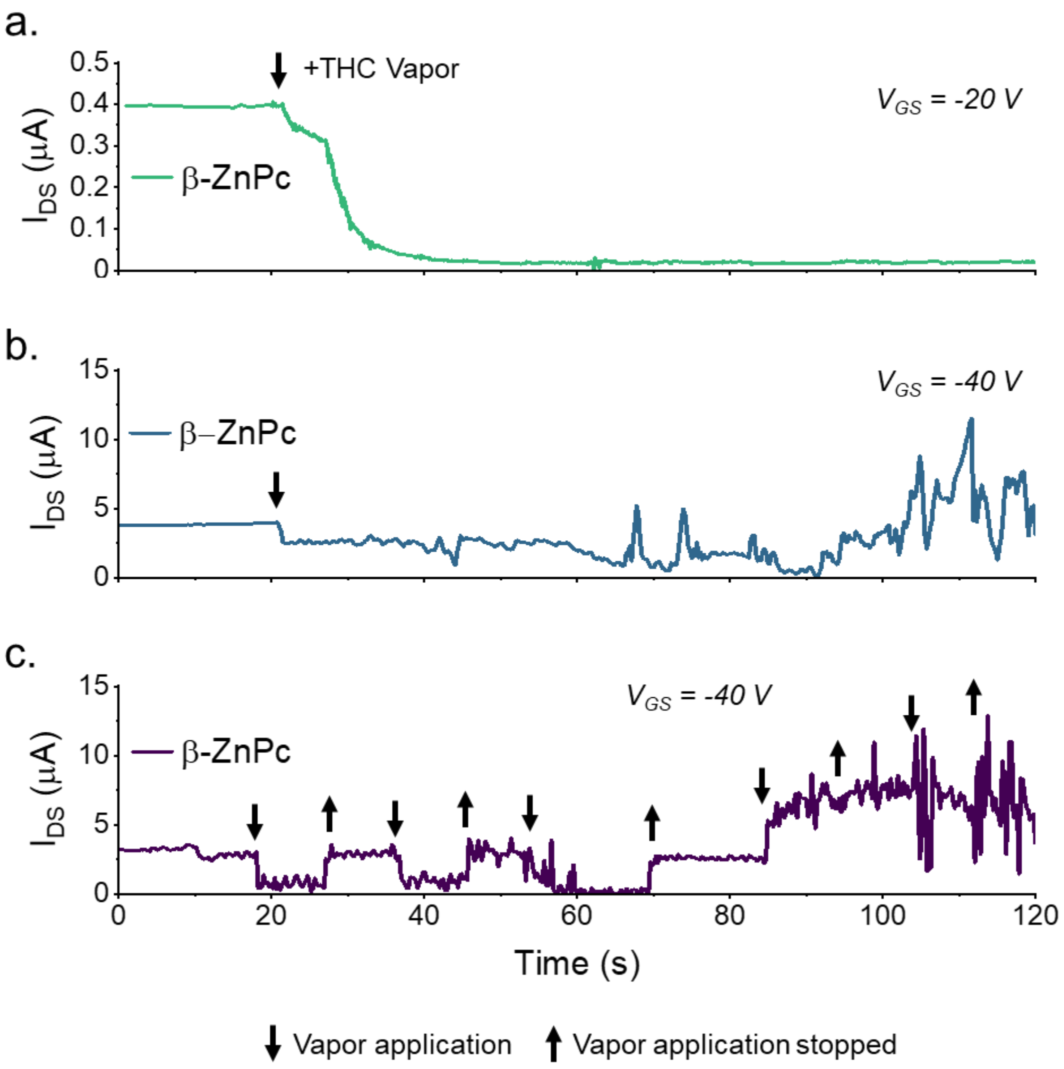


Figure S8. Continuous and periodic in-situ THC vapor exposure and detection. The effects of (a) continuous THC exposure when $V_{GS}$ = -20V, (b) continuous THC exposure when $V_{GS}$ = -40V, and (c) periodic THC exposure when $V_{GS}$ = -20V on β-ZnPc OTFTs. A $V_{SD}$ of -50 V was held and $V_{GS}$ was pulsed at a rate of 20 ms on 80 ms off over a period of 120 seconds. 400 ppb THC vapor was introduced to a 50 mL chamber.

## References

1. Hoshino, A., Takenaka, Y. & Miyaji, H. Redetermination of the crystal structure of α-copper phthalocyanine grown on KCl. *Acta Crystallogr. Sect. B Struct. Sci.* **59**, 393–403 (2003).

2. Yoon, S. M., Song, H. J., Hwang, I. C., Kim, K. S. & Choi, H. C. Single crystal structure of copper hexadecafluorophthalocyanine (F16CuPc) ribbon. *Chem. Commun.* **46**, 231–233 (2009).

3. Li, D. *et al.* Green synthesis and characterization of crystalline zinc phthalocyanine and cobalt phthalocyanine prisms by a simple solvothermal route. *CrystEngComm* **20**, 2749–2758 (2018).

4. Jiang, H. *et al.* Fluorination of Metal Phthalocyanines: Single-Crystal Growth, Efficient N-Channel Organic Field-Effect Transistors and Structure-Property Relationships. *Sci. Reports 2014 41* **4**, 1–6 (2014).
